# Beta lactam hypersensitivities

**DOI:** 10.1186/2045-7022-4-S3-P65

**Published:** 2014-07-18

**Authors:** Marie Eliane* Azoury, Noémie* Scornet, Stéphanie Delluc, Sandrine Delarue-Cochin, Cathy Nhim, Bernard Maillere, Richard Weaver, Nancy Claude, Delphine Joseph, Marc Pallardy

**Affiliations:** 1 UniverSud, Inserm UMR996, France; 2 UniverSud, UMR CNRS8076, France; 3 Platine Pharma service, France; 4 CEA, IbiTecS, Simopro, France; 5 Institut de Recherches Internationales Servier, France

## Background

Allergic reactions to drugs are often unpredictable and can lead to serious side effects such as anaphylactic shock. According to the hapten hypothesis, any drug with a molecular weight lower than 1000 daltons cannot induce an immune response by itself and must be bound to a protein. Antigen presenting cells, such as dendritic cells (DC), internalize hapten-protein conjugates and digest them into peptides bound with the drug. The latter are then presented on HLA molecules to drug-specific T cells inducing specific-drug immune response. Knowing that drugs provoke IgE mediated hypersensitivity reactions in treated patients, the CD4+ T-cell response to benzylpenicillin (BP) was investigated. The objectives of this study were to evaluate the frequency of naïve CD4+ T cells specific to BP and to identify BP-haptenized peptides responsible for T lymphocyte activation.

## Method

Since BP is known to bind covalently to proteins, such as Human Serum Albumin (HSA), HSA-BP bio-conjugates were synthesized at basic pH (pH=10.8). Seventeen BP binding-sites on HSA were then identified using mass-spectrometry, and 12 BP-haptenized peptides of 15 mer long potentially presented to T-cells via HLA class II molecules were identified and synthetized. Naïve CD4+ T cells from non-allergic donors were stimulated once a week with autologous DC loaded with HSA-BP or with peptide-BP to amplify respectively HSA-BP- or peptide-BP-specific T cells. Activation of specific CD4+ T cells was detected using interferon- EliSpot and their frequency was calculated using the Poisson distribution law.

## Results

The results of the CD4+ T-cell response to BP were as follows:

- Detection of HSA-BP-specific CD4+ T cells in 12 out of 13 tested donors with a mean frequency of 0.26 cells/million of circulating CD4+ T cells

- Identification of 17 binding sites of BP on HSA

- Specific naïve CD4+ T cells recognized at least 3 specific peptides, from HSA, haptenized by BP.

## Conclusion

This study showed the capacity of HSA-BP to be recognized by naïve T-cells from multiple healthy donors irrespective of their HLA typing and the possibility to identify BP-haptenized peptides involved in the allergic reaction to BP.

